# Supplementary material for: Comparison of the spatial and temporal distribution of cutaneous and mucosal leishmaniasis in the state of Rio de Janeiro between 2001 and 2011
Source: PLoS One. 2024 Dec 31;19(12):e0308298. doi: 10.1371/journal.pone.0308298 (PMC11687719; doi:10.1371/journal.pone.0308298)
Supplement: Attachment — Submitted filename: Reply to reviewers june 21, 2024 - Copiar.pdf [file pone.0308298.s004.pdf]

==

IMPORTANT: PLEASE DO NOT REPLY TO THIS EMAIL

If you are unable to complete any points that are requested in this email, please explain why in the 'Enter Comments' tab of the online submission form prior to re-submitting your manuscript. This will let us quickly assess your response and send your manuscript to an Academic Editor as soon as possible.

==
PONE-D-24-02709R1
Comparação da distribuição espacial e temporal das leishmanioses cutânea e mucosa no estado do Rio de Janeiro entre 2001 e 2011.
Dr Cláudia Maria Valete-Rosalino

Dear Dr. Valete-Rosalino,

We've checked your submission and before we can proceed, we need you to address the following issues:

1. Thank you for updating your data availability statement. You note that your data are available within the Supporting Information files, but no such files have been included with your submission. At this time we ask that you please upload your minimal data set as a Supporting Information file, or to a public repository such as Figshare or Dryad. DONE

Attached is the database. The data were reported by the Rio de Janeiro State Department of Health obtained from the Notifiable Diseases Information System (SINAN), from 2001 to 2011, subject to review.

Please also ensure that when you upload your file you include separate captions for your supplementary files at the end of your manuscript. DONE
As soon as you confirm the location of the data underlying your findings, we will be able to proceed with the review of your submission.

2. Please change the title in your online submission form in English. DONE

Comparison of the spatial and temporal distribution of cutaneous and mucosal leishmaniasis in the state of Rio de Janeiro between 2001 and 2011

3. Please amend the manuscript submission data (via Edit Submission) to include author Claudia Cristina Jardim Duarte. DONE

4. Please remove your figures/ from within your manuscript file, leaving only the individual TIFF/EPS image files. These will be automatically included in the reviewer's PDF DONE

5. We note that your manuscript is not formatted using one of PLOS ONE's accepted

file types. Please reattach your manuscript as one of the following file types: .doc, .docx, .rtf, or .tex (accompanied by a .pdf).
If your submission was prepared in LaTex, please submit your manuscript file in PDF format and attach your .tex file as “other.” DONE

6. Thank you for providing your data sources. Unfortunately, we are unable to see anything related to the data license used for your data sources. Please provide screenshots of evidence saying the data is open source or compliant with a CC-BY 4.0 license. DONE

We confirm that images 1, 2 and 3 presented in our submission were created by our authors specialized in geoprocessing, using the ARCHx program from ICICT - FIOCRUZ - Institute of Scientific and Technological Communication and Information - Fundação Oswaldo Cruz, they are maps prepared by our team with public data from the NOTIFICATION DISEASE INFORMATION SYSTEM - SINAN, therefore these are our figures and generated according to our results. Confirming then, that the maps do not come from any external location, website or public place that requires authorization. Attached is our database with the SINAN data that we used to prepare figures 1, 2 and 3, and the link below, as well as the capture made to prove that they are in accordance with the request of the reviewers and the magazine.

https://1drv.ms/x/s!AoJe38fYcml8g1Ic1WnD1DFV-vm6

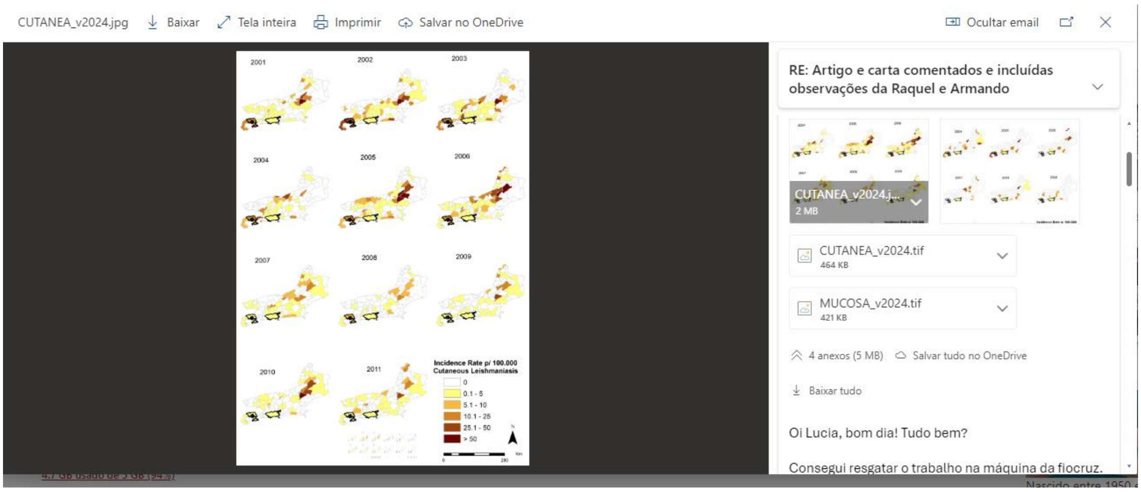

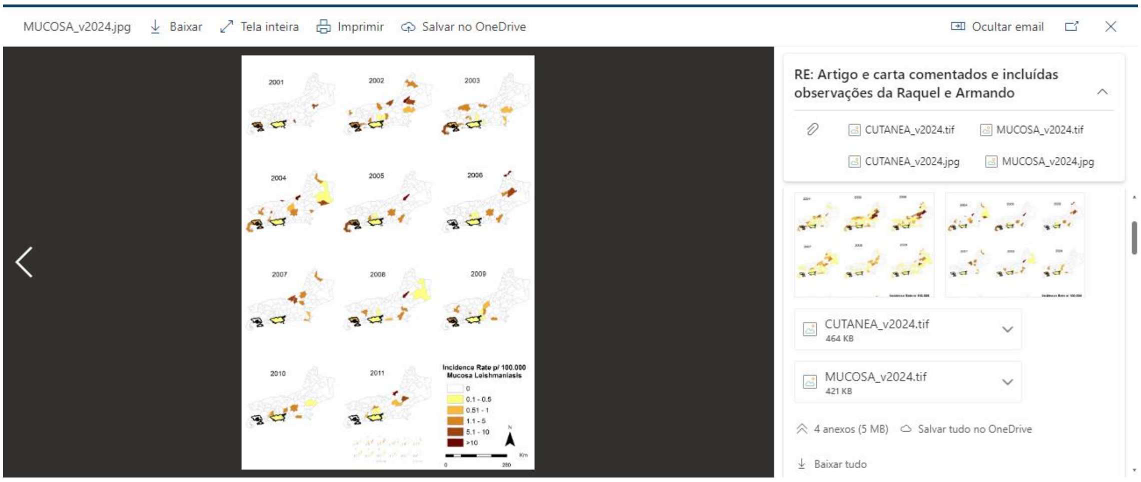
MUCOSA_v2024.jpg
Baixar
Tela inteira
Imprimir
Salvar no OneDrive
Ocultar email
2001
2002
2003
2004
2005
2006
2007
2008
2009
2010
2011
Incidence Rate p/ 100.000
Mucosa Leishmaniasis
0
0.1 - 0.5
0.51 - 1
1.1 - 5
5.1 - 10
>10
RE: Artigo e carta comentados e incluídas observações da Raquel e Armando
CUTANEA_v2024.tif
MUCOSA_v2024.tif
CUTANEA_v2024.jpg
MUCOSA_v2024.jpg
CUTANEA_v2024.tif
464 KB
MUCOSA_v2024.tif
421 KB
4 anexos (5 MB)
Salvar tudo no OneDrive
Baixar tudo

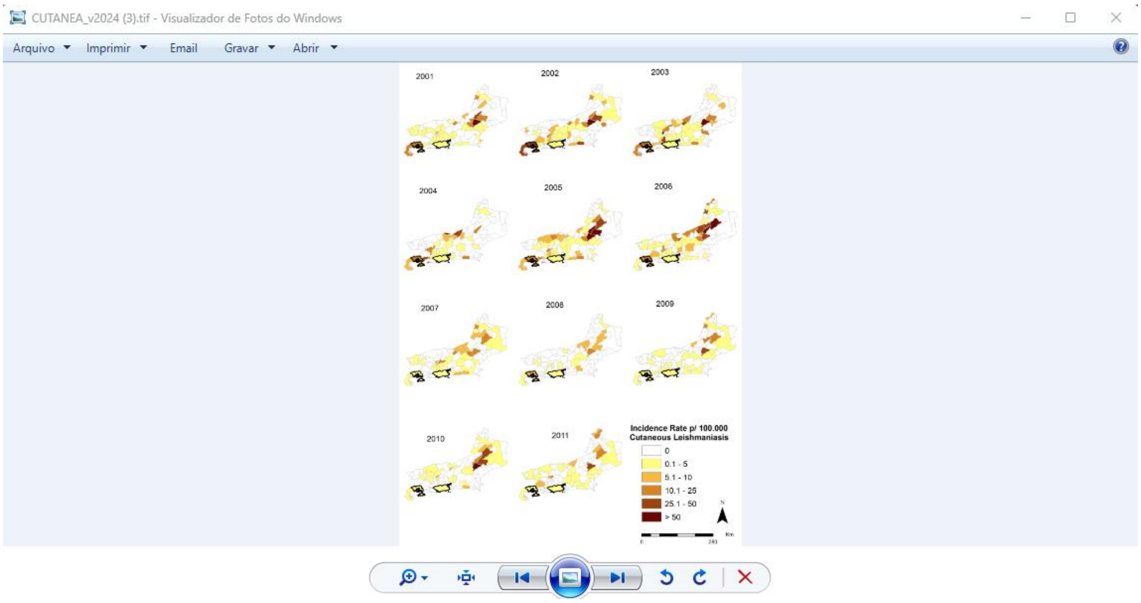
CUTANEA_v2024 (3).tif - Visualizador de Fotos do Windows
Arquivo
Imprimir
Email
Gravar
Abrir
2001
2002
2003
2004
2005
2006
2007
2008
2009
2010
2011
Incidence Rate p/ 100.000
Cutaneous Leishmaniasis
0
0.1 - 5
5.1 - 10
10.1 - 25
25.1 - 50
> 50

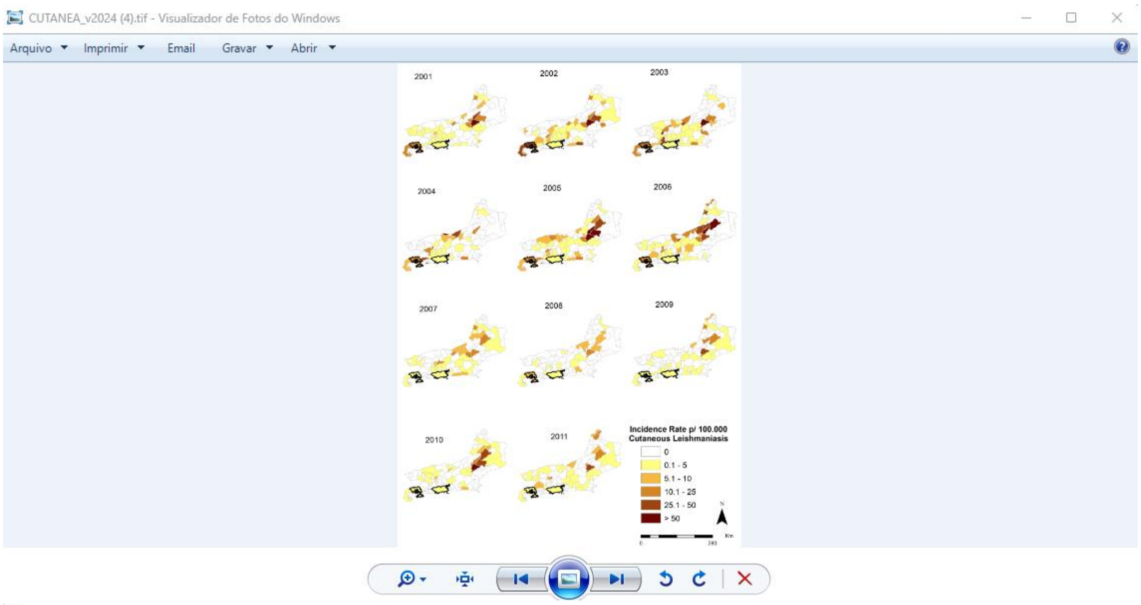

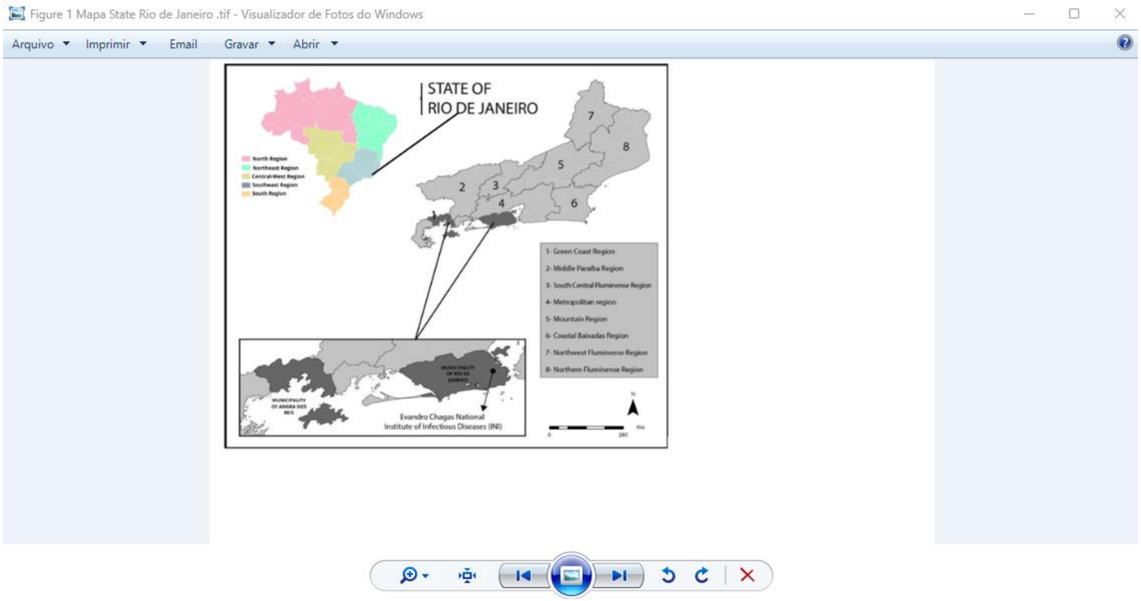

We've returned your manuscript to your account. Please resolve these issues and resubmit your manuscript within 21 days. If you need more time, please email the journal office at plosone@plos.org. We are happy to grant extensions of up to one month past this due date. If we do not hear from you within 21 days, we will withdraw your manuscript.

Please log on to PLOS Editorial Manager at https://www.editorialmanager.com/pone/ to access your manuscript. You will find your manuscript in the 'Submissions Sent Back to Author' link under the New Submissions menu. Be sure to remove your previous manuscript file if you are uploading a new file in response to these requests. After you've made the changes requested above, please be sure to view and approve the revised PDF after rebuilding the PDF to complete the resubmission process.

We are requesting these changes to comply with the PLOS ONE submission guidelines (https://journals.plos.org/plosone/s/submission-guidelines). Please note that we won't send your manuscript for review until you have resolved the above requests.

Thank you for submitting your work to PLOS ONE and supporting our mission of Open Science.

Kind regards,

Maidelyn R. Peregrin
PLOS ONE

In compliance with data protection regulations, you may request that we remove your personal registration details at any time. (Use the following URL: https://www.editorialmanager.com/pone/login.asp?a=r). Please contact the publication office if you have any questions.
