# Supplementary material for: Comparison of the spatial and temporal distribution of cutaneous and mucosal leishmaniasis in the state of Rio de Janeiro between 2001 and 2011
Source: PLoS One. 2024 Dec 31;19(12):e0308298. doi: 10.1371/journal.pone.0308298 (PMC11687719; doi:10.1371/journal.pone.0308298)
Supplement: Attachment — Submitted filename: Review.docx [file pone.0308298.s003.docx]

Overall, tt the objective is not clear what is the importance in compare the cases of whole state with specific municipalities, as Rio de Janeiro and Angra dos Reis, and also with the State. The methodology and presentation of model results are very confused, which compromises the interpretation of the study, as well as its purpose.

The introduction section is long and wordy.

Please, add line numbers.

Specific comments are below:

1. Regarding the sentences related to the reference 1:

“In the state of Rio de Janeiro, ATL is almost exclusively caused by this Leishmania species and it occurs in areas where phlebotomine vectors of peridomestic habits, such as Lutzomyia intermedia and Lutzomyia migonei, predominate.”

“Frequently, the infection occurs in domestic dogs, horses and eventually in domestic cats.”

Please cite the author(s) who found these informations. Although the Brazilian Ministry of Healt document is citing that data, there was not this Institution that did these studies and found these observations.

Please, cite references that found domestic dogs, horses and cats infected by the parasites. In the same way the authors who observed the predominant vectors species in Rio de Janeiro State. And please, pay attention in other citations regarding this reference “1”.

2. Regarding this phrase: “To our knowledge, only one autochthonous case of L. (Leishmania) amazonensis and two unusual cases of cutaneous leishmaniasis (CL) caused by L. (L.) infantum were described in the state of Rio de Janeiro.”

Please, cite the data up to the period in which this information is known.

3. About: “The long and variable incubation period could also hamper the epidemiological analyses of correlation between the occurrence or the introduction of new cases and the possible outbreak of secondary cases of CL and ML”

About “The long and variable incubation period”. In the previous phrase is citing an incubation period from 2 weeks to 2 months. Is this period long sufficiently to hamper the epidemiological analyses cited? Or are you referring to the period (12 years) of being infected after treatment, that is different of incubation period). Please, explain it better.

4. About the 4^th^ paragraph. It is not recommended to start phrases with acronym. The introduction section is too large, thus please evaluate whether describing the immunology of the disease is important data for your study. Just in case, cut some issues to reduce the introduction section. It seems
5. “… in the municipality of Rio de Janeiro, is responsible for the treatment of most ML patients notified in the RJ state.”

Define RJ acronym.

6. “Studies in that institution have revealed that, in the state of Rio de Janeiro, ….”

Please replace “…in the state of Rio de Janeiro” for in that State, because Rio de Janeiro is repeating close.

7. “…2005 (351 cases), and after that year there was a reduction of 85.07% in ATL incidence rate, decreasing from 1.44/100.000 inhabitants in 2004 to 0.20/100.000 inhabitants in 2013.”

Please explain it better. The year after 2005 is 2006, rather than 2004… This phrase is confused. In fact, the whole paragraph is confused, I suggest you resume the ideas, also because the introduction section is large.

8. “To date, there is no study on the spatial and temporal distribution of CL and ML cases. The objective of the present study was to compare the spatial and temporal distribution of CL cases with ML cases in the state of Rio de Janeiro and the municipalities of Rio de Janeiro and Angra dos Reis, between 2001 and 2011.”

This sentence is long and repeating terms. I suggest you clean up the text.

9. “160 neighborhoods, grouped into 33 administrative regions and seven district councils”.

“… the minimum absolute temperature registered in Angra dos Reis, since 1961, was 9.4°C on August 12, 1988, and the maximum absolute temperature was 39.3°C on February, 12, 1966.”

Look that these periods were a long time ago. Please evaluate if these informations are important for your study. It seems some data not relevant for your study and so the text is very long.

How many municipalities are in the state. This information is important, once they are focused on the analyzes. Are they 33? Please, specific it.

10. Cite “(Figure 1)” after the 1^st^ phrase of second paragraph of the methodology section, rather than at the end of that paragraph.
11. Fig 1

Legend of Figure: substitute “regions” for “Region”.

Brazil map: Please, add the limit of other Brazilian Regions, it seems that there are only the Region of Rio de Janeiro and one other….

Please add the limit of other states, at least in the highlighted Region, where Rio de Janeiro state is located. It seems that there are only the State of Rio de Janeiro and one other.

Down map: Please add grid of geographical coordinates	.

At the map is referring the City of Angra dos Reis and Rio de Janeiro and in the Legend of the Figure they are as Municipality. So, please, define if they are City or Municipality, because both terms mean different issues.

12. Population and ATL section

“… de Janeiro. (SESRJ), considering”. Here are point and comma.

Please, explain that you are calculating the incidence, because is not clear in this phrase why the “population” is considering here. In addition, explain how about the cases, their Municipalities are defined by Probably local of infection? Residence? Notification?

Are their years referring to the year of 1^st^ symptoms or of notification?

13. “… the spatial and temporal analysis of CL and ML occurrence”.

please mention the name of the spatial and temporal analysis here to inform the reader of the next steps to be explained. In addition, cite for what do you use the ArcGis software. Was it used to spatial and temporal analysis?

14. “All analyses were conducted for the state of Rio de Janeiro and for the municipalities of Rio de Janeiro and Angra dos Reis”.

How are you comparing data of state of Rio de Janeiro and for the municipalities of Rio de Janeiro and Angra dos Reis in the analyzes? Please explain it better.

If the State have 33 municipalities, are you comparing two municipalities with the other 31?

15. “A negative binomial regression model was used to assess the temporal dependency between the series.”

What are you meaning with “series”?

16. Model C: Regarding “rate for the year of study”. What are you meaning with “the year of the study”? Perhaps you can represent the years with *t* and *t-1* or *t-2*, when *t=year*… Could you please add the design of the models to be clearer? Perhaps using equations…
17. What are the independent variables of the models?
18. The dependent variables are the incidence per municipalities? For example: in model B the dependent variable is the incidence of ML_t-2_?

Without this both data and looking the results of models is difficult to understand the ideas of these analyzes.

I suggest you add in the results Section the table of model results, rather than the values in the body of the text.

19. “The model with smaller Akaike information criterion (AIC) was considered the best model.”

Is this considering for the choose of one of four models, or the choose of independent variables of best model for each one of four models?

20. “It is possible to observe that ML cases occurred in the same year or up to 5.5 years after the CL cases, with median of 1 year between both clinical forms.”

Please, explain better this observation, because it is not clear by just looking the Figures 2 and 3.

What are you meaning with “5.5 years”, once the figures are represented by complete years and not half a year.

Please add in the legends of Figures 2 and 3 that the incidence is per 100,000 habitants.

21. “Model C showed that ML rate (β1=6.9927, p=0.0231) tends to increase concomitantly with CL rate of the same year, even when controlled by the CL rate of the previous year (β2=0.3052, p=0.3039).”

This phrase is not clear, because the part “even when controlled by the CL rate of the previous year” had p value less than 0.05, that is not significant. Perhaps showing the table of model results (as suggested above) can elucidate the idea.

22. “Models A and C had good fit according to the deviance function… ”

I suggest you compare both models with statistical analysis, rather than just observing their values of p and AIC, as is in the text.

23. “In most localities the annual incidence rates of CL and ML increase concomitantly even when adjusted by the values of the incidence rates of CL of the previous year.”

Please, I suggest you specify the magnitude of this statement, just like "most" is how many percent (representing x% (x/x))? This can be mentioned in the result section and then make the statement more tangible.

24. “ATL distribution with CL incidence increase, followed by subsequent periods without notifications of this clinical form, as observed in some municipalities of the state of Rio de Janeiro, such as for example, the North and Northeast regions of the state, can be explained by the variation on the number of susceptible individuals in a location.”

How do you know the “variation on the number of susceptible individuals in a location”?

25. “In the state of Rio de Janeiro, from 2001 to 2013, ML represented 9.07% of all ATL cases, with a ratio of 10 CL: 1 ML.”

Is this 9.07% a percentage of all cases in Brazil in this period? Please explain it and add the reference. I also suggest you add the absolute numbers, such as 9.07% (x/x).

26. “The low susceptibility to the disease in a community or group (cases treated and healed, spontaneous healing, sub-clinical infections) occurs due to the acquisition of long lasting resistance of previously infected individuals.”

Please add reference, also for the follow phrase.

In addition, please, check if your examples of “low susceptible” are referring to “resistant population”, due to their immune development. When the population is compartmentalized according to dynamic disease (as demonstrated in the follow phrase of the text), the epidemiological concept is different for both susceptible and resistant individuals. The “low susceptible” individuals can be due to low number of them or individual characteristics, such as genetical immunity, sex, gender, and others….

27. “… where ATL would find an adequate and favorable environment for its development…”

Is the “disease ATL” that find adequate for its development, or is the dynamic cycle that could be maintained? It seems that the epidemiological terms are confused.

28. “…phlebotomine vectors are not reported in the surroundings of the Tijuca Massif, where the original arboreal vegetation was almost entirely substituted by reforesting with exotic species…”

Please add reference.

29. “The municipality of Rio de Janeiro displays a differentiated situation regarding other municipalities of the state of Rio de Janeiro.”

Please take of the second “Rio de Janeiro”. They are redundant.

30. “To date, no wild transmission cycle involving L. (V.) braziliensis was described in those areas, only the presence of infected domestic dogs, horses and cats, associated with the presence of Phlebotominae adapted to the modified environments.”

Please add a reference.

31. (Brito, 2016)

Please fit this reference according to PlosOne style, and also add it in the References section.

32. “It allowed the entry of susceptible population groups or those with ATL into modified environments, already with a high vector density, favoring and widening the transmission of the endemics in those areas, without the necessity of the existence of a specific reservoir.”

Please add the reference about “high density vector” at that area

33. “In the period from 2001 to 2013, INI/FIOCRUZ was responsible for the treatment of 27.4% of all CL cases, and of 76.2% of ML cases.”

Is this percentages regarding to the whole State or to the Country?

34. “the long time elapsed between the beginning of the symptoms and the diagnosis of mucosal manifestation of ATL”

What is this “long time”? Please, add a reference.

35. “Although the main agent involved in ML is L. (V.) braziliensis, other species have already been described, usually because of the proximity of the cutaneous lesion to mucous membranes: L. (L.) amazonensis, L. (V.) guyanensis and L. (V.) panamensis.”

Please, check if this reference is 25.
